# In-home occupational therapy for a patient with stage IV lung cancer: changes in quality of life and analysis of causes

**DOI:** 10.1186/s40064-015-0931-9

**Published:** 2015-04-01

**Authors:** Miyuki Imanishi, Hisao Tomohisa, Kazuo Higaki

**Affiliations:** Research Institute of Rehabilitation Sciences, Osaka Prefectural University, Habikino 3-7-30, Habikino-shi, Osaka Japan; Department of Psychiatry, Kyoto University Hospital, Shogoin-kawaracho 54, Sakyo-ku, Kyoto Japan; Department of Community Health, Osaka Prefectural University, Habikino 3-7-30, Habikino-shi, Osaka Japan

**Keywords:** End of life, Quality of life, Occupational therapy

## Abstract

**Introduction:**

We tracked and analyzed the changes in the quality of life (QOL) of a stage 4 lung cancer patient receiving occupational therapy at home.

**Case description:**

In a longitudinal study consisting of 4 evaluations over 9 months, a 66-year-old female with lung cancer was assessed using the Philadelphia Geriatric Center (PGC) Morale Scale and the 100-Point Satisfaction Scale. The QOL scores over time and factors influencing changes in these scores were analyzed.

**Discussion and evaluation:**

A histogram of QOL scores demonstrated a rapid increase followed by a mild decrease and then stable level. Interviews revealed the patient’s response to knowing her life expectancy, meeting a qualified occupational therapist, increasing her leisure activity, changing her family relationships and facing the prospect of death. We also confirmed that occupational therapy, such as writing letters or keeping a diary, reminded her of her late parents, hometown and childhood and helped her accept death.

**Conclusions:**

For a terminal lung cancer patient, meeting an occupational therapist to discuss fear or self-loathing improved QOL. Further, an active lifestyle played an important role in helping the patient accept death and lead a peaceful and stable life.

## Introduction

We conducted a follow-up survey of the changes in quality of life (QOL) scores for an occupational therapy patient with stage 4 lung cancer and analyzed the causes of these changes by conducting interviews and analyzing QOL scores.

The median survival of patients with unresectable stage 4 small cell lung cancer is 8–10 months. Even in cases that respond to anticancer drugs and radiation, average survival is approximately 20 months in cases with metastasis and 10 months in those without (Noda & Nishikawa 2000). Occupational therapists implement in-home therapy according to their patient’s wishes.

For patients entering old age who are experiencing psychological and social changes, the allocation of remaining time and acceptance of death is difficult. Although occupational therapy and other treatments at cancer centers in Japan have attempted to improve the QOL of cancer patients (Nishikori et al. 2011), the following questions remain unanswered: ‘What kind of QOL do terminal patients desire when staying at home?’ and ‘What does it mean to “be happy”?’ In Japan, an estimated 900,000 people will be diagnosed with cancer in 2015 (Takako & Tukuma 1999). Therefore, these are important issues that deserve careful consideration.

When measuring QOL scores in the elderly, the characteristics of their life style must be considered and their point of view from a psychological and social development perspective be adopted. In addition, the importance of evaluation from both subjective and objective standpoints has been reported (Koyano 2004a). The definition from the World Health Organization (WHO) in 1947 is regarded as the international standard in public health, and is as follows: “Health is a state of complete physical, mental, and social well-being and not merely the absence of disease or infirmity”. When faced with a life-threatening crisis, people experience a three-step process of isolation, transition and reconciliation (Van Gennep 1960). Upon facing a crisis, the qualities of finding strength to try and restore continuity, maintaining social interactions with friends and family, and maintaining religious faiths and other beliefs must come from within (Myerhoff & Simic 1978).

We therefore examined the application of occupational therapy in the final stages of life by following the path of a patient who transitioned from denial of disease and death to acceptance and desire to live their remaining life to the fullest.

## Methods

### (1) Study design

Qualitative research focusing on a patient and her family, friends and medical staff (12 people) was conducted. An ethnographic study was adopted and data obtained via direct interaction with the patient and surrounding individuals. A survey was conducted during visits with the cooperation of the patient, family, friends, the attending physician, nursing staff and other personnel (4 times over 9 months). A therapist visited the patient in her home twice a week (60 min per session) to perform occupational therapy. The first outcome measure was the Philadelphia Geriatric Center (PGC) Morale Scale. This scale was translated into Japanese, considered the standard reference values for community-dwelling elderly people and consisted of 17 questions, and its validity for elderly people living at home or in care facilities has been confirmed (Koyano 2004b). The second outcome measure was the 100-Point Satisfaction Scale. This is a simpler version of the Visual Analogue Scale of Happiness (VAS-H) that has been modified for elderly people (for example, explanations are delivered orally without the need for visualization), the results of which correlate with the VAS-H (Kobayashi 2002). The course of the patient’s QOL scores (chronological score graph), and interviews were then analyzed to investigate the main factors affecting QOL.

### (2) Sample case

A 66-year-old woman presented with symptoms of lower back pain, weakness and numbness of lower extremities and an intermittent limp. Soon after the onset of these symptoms, she experienced difficulty walking and was hospitalized. X-ray showed a shadow in the left lung and CEA levels were elevated. She was transferred to a specialist hospital due to possible bone metastasis. BS testing was administered and stage 4 lung cancer with metastasis in the lumbar vertebrae was diagnosed.

Initial treatment for bone metastasis with radiotherapy (30 Gy) was terminated. Gefitinib (Iressa) was then prescribed and a response observed. Oxycodone (Oxinorm) was prescribed for self-management of pain. The patient was discharged after one month of hospitalization and treatment.

Initial evaluation for rehabilitation revealed the following: paraplegia in the left hip joint flexion and internal rotation causing exhaustion, impaired sensation, declining strength, pain from the primary disease and a bedridden condition. However, cognitive function remained intact (MMSE28). The wish of the patient was to not be hospitalized again and of the family for prolongation of life.

### (3) Agreement and ethical approval

The patient required nursing care, had recently started using an in-home service and might have been unstable mentally due to physical and personal changes. Further, her participation in this study may have been an additional burden in terms of stress. Consideration was given to ethical concerns related to her privacy when cooperation was requested and when examinations and interviews were conducted. This study adhered to the agreement drafted by the ethics commitee of Osaka Prefectural University (Approval No. 2012-OT17).

## Results

Treatment progression was recorded every three months after baseline assessment, with four evaluations. The graph followed a course of a sudden rise, slight decline and a final equilibrium. Issues were discussed in interviews and were the basis for questions, as follows: shock of remaining time, meeting an occupational therapist, fulfilling leisure activities, changes in relationship with family and the topic of death. In addition, writing letters and journal entries evoked memories of her late parents, hometown and childhood, and encouraged statements demonstrating an acceptance of death. The patient’s condition, advice from the doctor and nurse, and the occupational therapist**’**s involvement are recorded below, focusing on the interviews at each evaluation. Two one-hour sessions for occupational therapy were conducted a week.

### Period 1: Isolation and rejection (start of therapy to 3 months)

#### Condition of patient

The patient was bedridden at initial intake, in a confused state, complained of pain, and exhibited despair, anger and impatience. The patient expressed frustration at being diagnosed with lung cancer despite never smoking and blamed secondary smoke from other members of her family. She stated that she would like to be able to use the toilet without assistance and would comply with rehabilitation.

#### Involvement of doctor and visiting nurse

The patient was given a one-year prognosis, provided that radiotherapy and chemotherapy were effective. The visiting nurse set the care goal of “living at least one more day”.

#### Involvement of the occupational therapist

##### Treatment plan: Training to improve physical behavior

At the initial intake, the patient’s wish was to go to the bathroom independently. As this was a period with large emotional fluctuations and repeated negative statements, the therapist took an educational approach and implemented activities of daily living (ADL) practice to improve body movement. After one month, the patient was able to move to a portable toilet. After two months, she was able to use a wheelchair to move about the house and use the toilet independently. After three months, she had recovered enough to use a T-cane to walk to the bathroom by herself.

### Period 2: Transition (3 to 5 months)

#### Condition of patient

Pruritus occurred as a side effect of medication and erythema affected her scalp. The patient distrusted medications, had general fatigue and voiced repeated dissatisfaction and complaints about the assistance of her family. She gradually began to talk about death from the fifth month, which may have stemmed from a belief of Buddhism.

The patient expressed acceptance of the inevitability of death but not the disease. She was satisifed with her progress regarding rehabilitation and reflected on what her family was thinking. She also confided her feelings upon receiving her diagnosis and frustration at not noting her symptoms earlier.

#### Involvement of doctor and visiting nurse

The doctor reported the tumor marker results at each examination. He advised the patient to undertake activities like taking family trips that create good memories while levels were stable. He also mentioned that after the examination, friends and family could send emails inquiring about the marker values. The nurse then explained that the patient should try not to worry about side effects from drugs, such as hair damage, and encouraged her to keep working on her rehabilitation to improve her walking ability.

#### Involvement of occupational therapist

##### Treatment plan: Training to achieve independence in ADL

Although the patient continued to complain that her body was in bad condition, she remained optimistic and worked hard in training. Suggestions were made regarding “balancing activity and rest”. Upon discovering that the patient wrote a diary entry every night before her illness, the therapist encouraged the patient to resume writing in her diary and writing letters. At one point, the patient again recalled learning of her diagnosis. From this point, the therapist**’**s involvement changed from an instructive style to a patient-centred approach based on the Canadian Occupational Performance Measure (COPM). The therapist only spoke in response to questions she was asked and listened closely to what the patient had to say.

### Period 3: Transition and start of acceptance (5 to 8 months)

#### Condition of patient

The patient’s lifestyle gradually reached a stable rhythm. She was also more positive and had fewer complaints of dissatisfacion with her family. When there was a visitor, she sometimes spoke of her past memories.

The patient stated that loneliness was a contributing factor that hampered her recovery. She stated the benefit of open and honest communication and desire to no longer take medications with strong side effects. Further, she also expressed acceptance of death.

#### Involvement of doctor and visiting nurse

Although the doctor recommended prescribing a different anticancer drug, the patient requested a more specific explanation of the side effects and decided not to take it. This conflicted with the family**’**s hopes for prolongation of life, but they respected her decision. At the same time, she planned a final trip to her home town. With the high risk of fracture due to bone metastasis, the nurse urged her not to go. However, the patient self-managed her painkiller usage and took a trip to her hometown more than 10 hours away.

#### Involvement of occupational therapist

##### Treatment plan: Occupational therapy program based on reading and writing activities

In response to reports of the patient’s excessive activity, such as staying up all night writing letters to her family, the occupational therapist paid attention to vital signs and reorganized the therapy program as necessary, such as replacing rehabilitation time with relaxation.

### Period 4: Reconciliation and acceptance (8 months to end of life)

#### Condition of patient

Although the patient stopped complaining of bodily pain, she self-managed these symptoms with painkillers. She began to talk of her love for her late parents, memories of childhood and discussed religion and how her life after witnessing the passing of her grandfather and patients. Casual conversation also increased and expanded into calm interactions.

The patient expressed gratitude for being able to cook and eat independently and stated that she now had an appetite. She was content with her present quality of life and remained accepting of death. In addition, she maintained that she did not want to take medications with strong side effects that were detrimental to her quality of life.

#### Involvement of doctor and visiting nurse

As the patient decided to stop treatment due to her declining QOL, her regular visits to the hospital were replaced with house calls by a local doctor. The doctor discussed pain, sleep and bowel control issues with the patient and implemented treatment. The visiting nurse stopped providing services due to the patient’s desire to spend her time entertaining visitors.

#### Involvement of occupational therapist

##### Treatment plan: Counseling sessions on the acceptance of death

**Figure 1:**
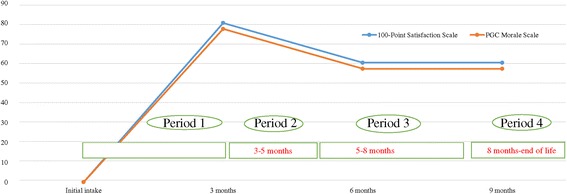
**The path of client A’s QOL scores.**

In response to religious discussions, the therapist listened closely in silence and nodded in agreement. For simple everyday conversation, the therapist assumed the role of a socially inexperienced person asking to be taught. However, this did not mean that the patient felt no pain and loneliness. The therapist sent a clear message to the patient that she should feel comfortable sharing her feelings (Figure 1).

## Discussion

At 11 months and 21 days after the start of occupational therapy, the patient’s condition suddenly worsened, and after 2 days in a lethargic state, she passed away at the age of 67 in her home.

The path of the patient after hearing her prognosis started with an isolation phase, which was marked by a desperate hope to escape her reality. She next entered the first transitional period and began to face the concept of death. The patient valued every day as special and irreplaceable. Even when there were inconvenient and difficult experiences, she went about her daily life, marking the reconciliation phase.

Although counselling and treatment are important aspects of occupational therapy (Sumsion 1999), accurate evaluation of the stage of the patient’s psychological and social development and modifying the treatment relationship appears helpful in accelerating the patient’s development and improving QOL. This is particularly relevant if treatment sessions are long, become a part of the patient’s daily life, and the visits of the therapist become house calls.

In the present case, the occupational therapist developed the patient’s therapy while continuing to listen closely in the initial period, during which the patient struggled with psychological conflicts. Once a favorable rapport had been built, the therapist changed the approach to a more cooperative one, providing frequent opportunities for discussion with the patient about the occupational therapy program. In the final stage of therapy, the patient gave maximum priority to QOL improvement and was able to live the life that she wanted.

In the final stage, the patient met the occupational therapist—who discussed her anxiety and self-loathing—and this meeting became an opportunity for the patient to consider QOL improvement for herself, which became a major factor in actual QOL improvement. Occupational therapy supported the patient’s life, and provided acceptance of death and a sense of peace and hope. The occupational therapist also displayed a consistent stance to the patient.

The patients’s improvement in QOL with in-house occupational therapy was due to the continuing support for her independence. This might have been due to the occupational therapist remaining consistent and sincere with the patient throughout the process.

### Key messages

- Occupational therapy improved the QOL of a terminal lung cancer patient.
- An active lifestyle played an important role in helping the patient accept death.

## References

[CR1] Kitagawa T, Hideaki T (1999). Cancer Statistics “Japan Cancer Morbidity Prediction”. Cancer Statistics.

[CR2] Kobayashi N (2002). Evaluation of Subjective QOL in Elderly-modified PGC Morale Scale and the Degree of Satisfaction in the Scale of 100 Points. Soc Gerontol.

[CR3] Koyano W (2004). Current QOL Research in Social Gerontology and Issues. Health Care Sci.

[CR4] Koyano W (2004). QOL Studies in Japanese Social Gerontology. J National Inst Public Health.

[CR5] Myerhoff B, Simic A (1978). Life’s Career-aging.

[CR6] Nishikori M, Mitikawa M, Tateyama K, Higaki K (2011). Occupational Therapy Questionnaire Survey for Cancer in our Country. J Rehabil Health Sci.

[CR7] Noda K, Nishikawa Y (2000). Irinotecan plus Cisplatin Compared with Etoposide plus Cisplatin for Extenshive Small-Cell Lung Cancer. N Engl J Med.

[CR8] Sumsion T (1999). Client-centered Practice in Occupational Therapy: A Guide to Implementation.

[CR9] Van Gennep A (1960). The Rites of Passage.

